# The Abundance and Pollen Foraging Behaviour of Bumble Bees in Relation to Population Size of Whortleberry (*Vaccinium uliginosum*)

**DOI:** 10.1371/journal.pone.0050353

**Published:** 2012-11-27

**Authors:** Carolin Mayer, Denis Michez, Alban Chyzy, Elise Brédat, Anne-Laure Jacquemart

**Affiliations:** 1 Earth and Life Institute, Catholic University of Louvain, Louvain-la-Neuve, Belgium; 2 Zoology Lab, University of Mons, Mons, Belgium; Royal Holloway University of London, United Kingdom

## Abstract

Habitat fragmentation can have severe effects on plant pollinator interactions, for example changing the foraging behaviour of pollinators. To date, the impact of plant population size on pollen collection by pollinators has not yet been investigated. From 2008 to 2010, we monitored nine bumble bee species (*Bombus campestris, Bombus hortorum* s.l., *Bombus hypnorum, Bombus lapidarius, Bombus pascuorum, Bombus pratorum, Bombus soroensis, Bombus terrestris* s.l., *Bombus vestalis* s.l.) on *Vaccinium uliginosum* (Ericaceae) in up to nine populations in Belgium ranging in size from 80 m^2^ to over 3.1 ha. Bumble bee abundance declined with decreasing plant population size, and especially the proportion of individuals of large bumble bee species diminished in smaller populations. The most remarkable and novel observation was that bumble bees seemed to switch foraging behaviour according to population size: while they collected both pollen and nectar in large populations, they largely neglected pollen collection in small populations. This pattern was due to large bumble bee species, which seem thus to be more likely to suffer from pollen shortages in smaller habitat fragments. Comparing pollen loads of bumble bees we found that fidelity to *V. uliginosum* pollen did not depend on plant population size but rather on the extent shrub cover and/or openness of the site. Bumble bees collected pollen only from three plant species (*V.* *uliginosum, Sorbus aucuparia* and *Cytisus scoparius*). We also did not discover any pollination limitation of *V. uliginosum* in small populations. We conclude that habitat fragmentation might not immediately threaten the pollination of *V. uliginosum*, nevertheless, it provides important nectar and pollen resources for bumble bees and declining populations of this plant could have negative effects for its pollinators. The finding that large bumble bee species abandon pollen collection when plant populations become small is of interest when considering plant and bumble bee conservation.

## Introduction

The destruction and fragmentation of formerly continuous plant communities is considered to be one of the major threats for plant-pollinator interactions [1]. Besides mere reduction of habitat size, fragmentation also implies increased isolation, edge effects, and reduced connectivity among different patches which could further enhance or alter the negative effects on plants and animals [2], [3]. Within a pollination network, the loss of some dominant species can lead to concurrent decline or extinction of associated species [4]. Typically, plants may suffer from a reduced abundance and diversity of pollinators resulting in limited pollen transfer [5], [6] and reduced reproductive success in fragments [7], [8].

Pollinators, such as bees, can be affected by resource limitation and competition for food may increase in small habitat fragments [9], [10]. Certain life history traits may render some pollinator species more sensitive to habitat loss: specialized species for example might not be able to shift to alternative host plants [11]. Large bee species require larger amounts of pollen to feed their larvae and would leave sites with low flower abundance first [12]–[14]. On the other hand, smaller bee species unable to cover long distances [15], [16] might be incapable of re-colonising fragments and could suffer more severely from increasing isolation of habitat fragments [3].

The majority of studies investigating effects of habitat fragmentation on plant communities concentrate on single plant species during only one flowering season [17], [18] and typically focus on abundance and diversity patterns of flower visitors [19]. In the few cases where pollinator behaviour has been considered, only variables such as number of inflorescences and/or flowers per inflorescence visited, time spent per flower or search duration are analysed [8], [20]–[22]. Other aspects of foraging behaviour related to diet breadth or food resource availability are still poorly investigated, especially for bees [19], [23].

In this three-year study, we studied the effects of population fragmentation of *Vaccinium uliginosum* (whortleberry, Ericaceae) on the abundance, species richness and behaviour of its main pollinators, bumble bees, and how this affected plant reproductive success. *Vaccinium uliginosum* is a deciduous perennial shrub growing in bogs and is rare and threatened by land use and climatic changes due to this habitat preference [24]. It is present in Belgium only at higher altitudes, such as the Upper Ardenne (>500 m). Here, peat bogs have largely been destroyed to give way to spruce (*Picea abies*) plantations since the middle of the 19th century. Today, highly fragmented and isolated patches remain [25], many of which are under protection and subject to conservation efforts.

In up to nine sites we investigated whether: (1) abundance and diversity of bumble bees are reduced in small plant populations; (2) other fragmentation characteristics, such as spatial isolation or enclosure by spruce plantations, influence abundance and species richness of bumble bees; (3) foraging behaviour of bumble bees changes as population size of forage plants decreases; (4) bumble bees show reduced fidelity to *V. uliginosum* in small populations; (5) *V. uliginosum* suffers from pollination limitation and reduced seed set in small populations.

## Methods

### Plant Species

**Figure 1:**
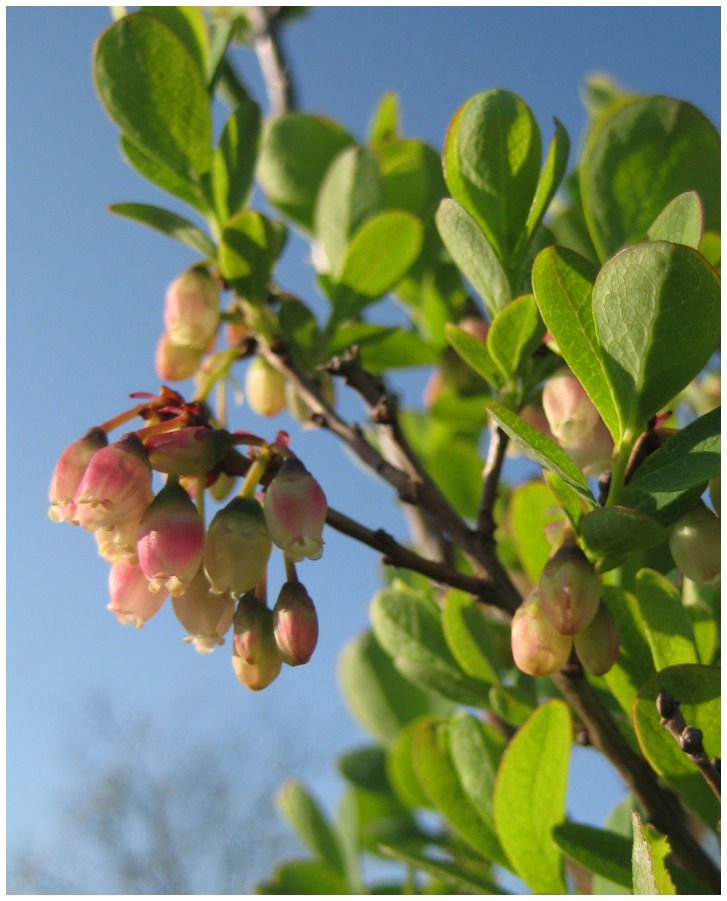
A flowering shoot of *Vaccinium uliginosum* (photograph by C. Mayer).

*Vaccinium uliginosum* ssp. *uliginosum* L. is a perennial shrub which propagates by horizontal subterranean rhizomes. It has a circumboreal distribution growing on acid, poorly drained and wet soils and prefers habitats like wet heaths and bogs [26]. In Ardenne, the flowering season starts in late spring (mid May) and lasts for about 25 days [27]. The subglobose flowers are pale pink to white and droop from the terminal shoot (Fig. 1). They are visited by a variety of bumble bees and syrphids, but also solitary and honey bees [27].

### Study Sites

Four core sites with *V. uliginosum*, with population sizes ranging from 81 m^2^ to over 30000 m^2^, were studied for three consecutive years from 2008 to 2010 (Table 1). Five additional sites were observed for two entire days during May and June 2010. These represent all remnant populations on the Plateau des Tailles in the Upper Ardenne, Belgium. They are all restricted to an altitude between 550 and 605 m, comprising a total area of about 3825 ha. Study sites were mapped with the help of a GPS (Magellan sportrack pro) and georeferenced images of Google Earth. The following spatial fragmentation characteristics of the sites were calculated with MapInfo professional version 5.5: we refer to ‘population size’ (“plant surface area” in Table 1) as the sum of the area covered by *V. uliginosum* shrubs. This value reflects actual population size more accurately than the entire “surface area of the study site”, which includes between-shrub patches covered mainly with grasses (*Molinia caerulea*). At the study sites the density of *Vaccinium*-shrubs can vary appreciably: only “Wé des Pourceaux” and “Fange aux Mochettes” had an almost completely closed shrub layer (“shrub cover”  = 100%; Table 1). All other sites are comprised of more or less scattered patches of *V.* *uliginosum*. Flower density per m^2^ on the other hand does not differ significantly between different sites and years [28]. The “site perimeter” is the total length of the edge of the study site. “Edge density” was calculated as site perimeter divided by the surface area of the study site following Holzschuh et al. [29]. The area of the surrounding bog (“bog surface area”), gives an indication of the size of the potential habitat that would be suitable for the plant. The proportion of the “surrounding forest” further indicates whether a site lies within a large open landscape or is enclosed by forest and spruce plantations, which could further reduce patch connectivity (like Wé des Pourceaux, Fange aux Mochettes; Table 1). “Isolation” was measured as the linear distance (from centre to centre) between two neighbouring sites [30].

**Table 1:** Characterization of *Vaccinium uliginosum* populations arranged by increasing plant surface area (study sites in italics were observed 2008–2010, others only in 2010).

Study site	Coordinates	Plant surface area (m^2^)	Surface area of the study site (m^2^)	Site perimeter (m)	Edge density (m^−1^)	Shrub cover (%)	Bog surface area (m^2^)	Surrounding forest (%)	Isolation (m)	Altitude(m)	Habitat
*Grande Fange*	*50°14′40′’N 5°46′45′’E*	*81*	*213*	*69*	*0.32*	*38.2*	*1 295 000*	*62*	*1 130*	*557*	Open, continuous to patchy; Lithalsa*, part of large fen
*Wé des Pourceaux*	*50°14′40′’N 5°46′40′’E*	*97*	*97*	*48*	*0.50*	*100.0*	*172*	*100*	*610*	*602*	Closed, continuous; Lithalsa*, part of Sacrawé reserve
Chamfa	50°13′07′’N 5°47′57′’E	193	9 059	437	0.05	2.1	446 700	56	1 130	563	Open, patchy, wet heathland
Robièfa	50°15′32′’N 5°41′59′’E	359	1 407	167	0.12	25.5	6 859	100	1 260	545	Closed, patchy, wet heathland
Pisserotte	50°13′10′’N 5°47′02′’E	394	10 500	403	0.04	3.8	180 600	96	1 010	572	Closed, patchy, wet heathland
Nazieufa	50°15′18′’N 5°43′00′’E	670	14 560	506	0.04	4.6	358 800	58	1 260	606	Open, patchy, wet heathland
Massotais	50°14′13′’N 5°45′34′’E	719	42 660	880	0.02	1.7	1 180 000	54	850	604	Open, patchy, bog with lithalsas*
*Fange aux Mochettes*	*50°13′22′’N 5°40′54′’E*	*6 917*	*6 917*	*399*	*0.06*	*100.0*	*66 270*	*100*	*4 190*	*602*	*Closed, continuous, ombrotrophic bog*
*Sacrawé*	*50°14′40′’N 5°45′32′’E*	*31 580*	*51 400*	*1 094*	*0.02*	*61.4*	*548 100*	*78*	*610*	*600*	*Open, continuous to patchy, wet heathland*

“Plant surface area” is the sum of the area covered by *V. uliginosum* shrubs. “Surface area of the study site” includes between shrub patches, “site perimeter” is the length of the edge of the study site. “Edge density” is the perimeter divided by surface of the study site. “Shrub cover” is “Plant surface area” per “surface area of the study site”. “Bog surface area” refers to the surrounding habitat, which is partly surrounded by forest plantations (“Surrounding forest”). “Isolation” is the distance to the nearest neighbouring study site. *Lithalsas are ramparted depressions covered with floating bog vegetation. Further explanations see text.

### Visitor Abundance, Diversity and Behaviour

In May 2008 and 2009, flower visitors were recorded during peak flowering for one entire day (6.00 a.m. to 9.00 p.m.) in each of the four core populations. Compared to single time slots spread over several days, this method maximizes the probability of observing all potential flower visitor species at a study site. It further minimizes the effects of variable weather conditions, a common risk of field work in Belgian springs. To achieve a larger sample size of populations, we conducted observations at five additional study sites in 2010. Different observers recorded flower visitors at all nine sites at the same time on two dates (May 25^th^ and June 4^th^; note that one small site, Wé des Pourceaux, was not yet flowering in May). The total observation time was 298 h.

Insect visitors foraging on *V. uliginosum* were collected with an insect net from a 10 m^2^ plot of continuous shrub cover for 20 minutes each hour on the same plot. These insects were identified and then released [31]. Determination in the field was made to operational taxonomic units (OTU) that cluster similar species that are impossible to distinguish under field conditions. To evaluate species diversity within each OTU, we collected about 100 specimens in 2008 and 2010 after flower observations. For some analyses, bumble bee species were grouped according to their size into two categories: small species with an average queen length <20 mm and wingspan <35 mm (Table 2) and large species with queens ≥20 mm long and a wingspan ≥37 mm after Benton [32]. We noted sex and caste of bees and whether they foraged for pollen or nectar, i.e. whether they carried corbicular pollen loads. Pollen analyses confirmed that all but three examined bees with corbicular pollen had collected *Vaccinium* pollen (n = 127; see results below).

**Table 2:** Numbers of individual bumble bees recorded visiting *Vaccinium uliginosum* flowers at different study sites during 298 h of observation.

	Species:	Large sized			Small sized					
		*B. terrestris* OTU	*B. lapidarius*	*B. vestalis* OTU	*B. pratorum*	*B. pascuorum*	*B. hortorum* OTU	*B. hypnorum*	*B. campestris*	*B. soroensis*
	Size; wingspan:	20; 40	22; 38	21; 37	16; 30	17; 32	19; 35	18; 35	17; *	16; 30
**Study site**	**Year**									
Grande	2008				3	1				
Fange	2009	4			15	33	1			
	2010 May	8	1	2	2	25	1			
	2010 June	4	3	9	9	39				2
Wé des	2008			2	17			2		
Pourceaux	2009	3	1	4	28	4		4		
	2010 June	11	3	5	15	11				
Chamfa	2010 May	2	2	1	4	3				
	2010 June	4	2	14	9	9		3		
Robiefa	2010 May	12	7		10	8				
	2010 June	4	1	8	5	5	1	4		
Pisserotte	2010 May	7	4	1	19	3		2		
	2010 June	17		24	18	13				
Nazieufa	2010 May	19	11		12	4				
	2010 June	5			10	3				
Massotais	2010 May	18	2	1	5	20				
	2010 June	10				1	3			
Fange aux	2008	16	2		28	2		3		
Mochettes	2009	3	1	2	42	9	38	7		
	2010 May	19		2	34	13	4	2		
	2010 June	8		7	87	5	4	10		
Sacrawé	2008	19	27		15	10	7	3		
	2009	87	73		56	9	4	9	1	
	2010 May	19	8	1	3	1	2	4		
	2010 June	62	23	5	24	11	4	5	1	
**Total**		**361**	**171**	**88**	**470**	**242**	**69**	**58**	**2**	**2**

Populations descend from small to large. Species are grouped into size classes after Benton [32]. *wingspan unknown.

### Visitor Fidelity

Several studies have shown that field records alone are not a reliable method to identify pollen-host associations because they rarely distinguish between pollen and nectar foraging [33], [34]. However, accurate quantitative estimates of pollen-host use and fidelity in bees can be achieved by pollen analyses of corbicular pollen loads [35]. We sampled pollen loads in 2008 and 2009 from bumble bee workers of different species (*B. hortorum* s.l., *B. hypnorum, B. lapidarius, B. pascuorum, B. pratorum, B. terrestris* s.l.) in the four core populations. After female bees had been immobilized in a bee marking cage, the pollen packets were gently removed with a toothpick. Pollen loads were acetolyzed [36] and from each sample, about 400 randomly chosen pollen grains were identified by light microscopy at 400× magnification. In this study, we present detailed results for *Bombus pratorum* for which we retrieved the largest number of samples from all four core populations (54 out of 127). We further pooled the pollen loads of all species and grouped them according to their origin into “small” (“plant surface area” <100 m^2^; n = 32) and “large” (n = 95) populations. For a second model they were classified according to openness of the site and shrub cover into “open+patchy” (n = 76) and “closed+continuous” (n = 51) populations. “Closed+continuous” populations were those completely surrounded by spruce plantations with a shrub cover of 100% (Fange au Mochette and Wé de Pourceaux, Table 1).

### Pollen Limitation of *Vaccinium uliginosum*

To investigate a possible impact of fragmentation on plant-pollinator-interactions and the reproduction of *V. uliginosum*, we conducted experiments with supplemental hand pollination for the four core populations in May 2009. To reduce potential geitonogamous pollination, pollen from several different shrubs in the same population was collected on a microscopic slide with the help of a tuning fork and mixed before application. On average, five flowers clustered on one twig (min = 2, max = 13) were hand-pollinated with outcross pollen (“hand-pollinated”). Five additional flowers on the same plant were marked and left to natural pollination (“open-pollinated/same plant”). Another five flowers on a different plant (located within 1–2 m) were marked and left to natural pollination (“open-pollinated/different plant”) to account for resource allocation effects within one plant. In the large populations (Sacrawé and Fange aux Mochettes) we repeated this treatment 15 times (with treated individuals ≤6 m apart). In the small populations (Grande Fange and Wé des Pourceaux) only ten repeats were feasible. At the Grande Fange site it was impossible to find control plants (“open-pollinated/different plant”) since this population consists of one patch containing possibly only one or two clones. Eight weeks later, ripe fruits were collected and seeds and ovules were counted to calculate seed set as the number of viable seeds per ovule. Seeds were categorised according to their size and form as ‘viable’ (length ≥1.5 mm, and smooth ovoid shape) or ‘aborted’(length <1.5 mm, coarse irregular shape). Viability was verified for 30 seeds, from each of these two groups, by staining them with a solution of 1% Tetrazolium following the protocol of Kearns and Inouye [37].

### Statistical Analyses

First, we tested for intercorrelations among the different spatial variables (Table 1) with Spearman Rank Correlations. Surface area of the study site, perimeter measurements and edge density were excluded from further analysis, since they were highly correlated with plant surface area, considered to be the best measure of plant population size (*r* ^2^ ranging from 0.75–0.98, *P*<0.05). The remaining fragmentation characteristics were plant surface area, bog surface area, isolation and the proportion of surrounding forest. These were defined as influencing (fixed) factors and related to the different response variables by estimating generalised linear mixed models (GLMM). We pooled our observations across all sites and dates, resulting in 25 statistically non-independent data points that had to be analysed carefully to avoid the criticism of artificially inflating the degrees of freedom (i.e. pseudoreplication) [38]. “Pseudoreplication” is defined as the misanalysis or misinterpretation of replicates that are not statistically independent such as the repeated observation of the same subjects (populations in our case) [39]. GLMMs however, correctly analyse such hierarchically structured and unbalanced data sets as ours and effectively eliminate the statistical problem of pseudoreplication [38], [40]. This was achieved by including the different observation dates and the study sites as random factors in the models, where date was nested within study site [41]. The following response or dependent variables were calculated per site and observation date: total number of bee individuals and species visiting *Vaccinium* flowers, Shannon diversity index, proportion of small and large bumble bee species, proportion of bees collecting pollen, proportion of small and large bee species collecting pollen (i.e. within the group of small/large species, the percentage of individuals with corbicular pollen). Again with GLMMs, the fidelity of bumble bees to *Vaccinium* according to population characteristics was investigated by analysing the percentage of *Vaccinium* pollen in the loads pooled for all bumble bee individuals (dependent variable, n = 127). Fixed factors in these models were the mentioned classes of population size (“small” or “large”) and proportion of surrounding forest and shrub cover (“open+patchy” or “closed+continous”). Here, the different bumble bee species, as well as the study site, were included as random factors. For all GLMMs, we used a negative binomial error distribution for count data, with a log-link function and Laplace likelihood approximation, which effectively reduced overdispersion in variance of the data. For proportional data, best model adaptation was achieved by using a beta distribution with a logit function and residual penalized likelihood approximation [42]. Goodness of fit was further improved when fixed factors were log_10_ transformed. The significance of each of the fixed effects specified in the model was tested with "Type III Tests of Fixed Effects". We used Kruskal-Wallis and Mann-Whitney-*U* tests to examine effects of population on pollen loads of *B. pratorum* and to compare seed set of fruits from different pollination experiments. If not indicated otherwise, average values are presented as mean±standard deviation. GLMMs were estimated with SAS 9.2 (“Proc GLIMMIX”; SAS Institute Inc., Cary, NC, USA); other analyses were computed with R 2.13.0 [43].

## Results

### Visitor Abundance, Diversity and Behaviour

During three years of observation, we recorded 1463 bumble bees from 9 OTUs (representing 13 species) which corresponded to 50.7% of all insects recorded (n = 2879, Table S1). Other flower visitors were mainly syrphid flies (43.4%), six species of solitary bees (2.8%, especially *Andrena* spp.), and honey bees (*Apis mellifera*, 1.9%). All other groups (Sarcophagidae, Lycaenidae and Vespidae) were encountered at very low frequencies amounting to less than 1% of all flower visiting individuals.

**Figure 2:**
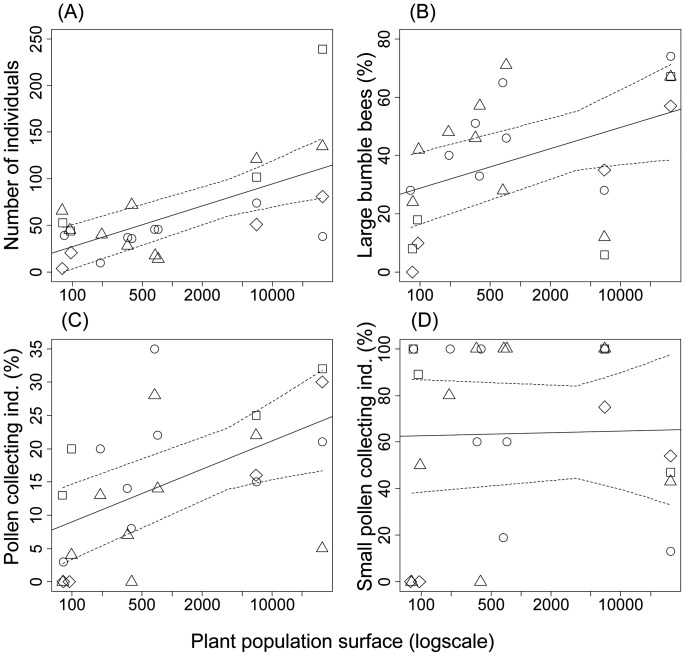
Different bumble bee variables plotted against plant population size. Number of bumble bee individuals (A), proportion of large bumble bee species (B), proportion of bumble bees collecting pollen (C) and proportion of small bumble bee species collecting pollen (D). Data points are jittered on × axis with 2008: diamond, 2009: square, May 2010: circle, June 2010: triangle. Solid and dashed lines: regression line with 95% CI-bands.

In general, the number of bumble bees observed differed tremendously across days and sites: between 4 and 239 bumble bee individuals (58.4±49.8) and between 2 and 8 different OTUs (5.2±1.5) were recorded (Table 2). We found significantly higher numbers of bumble bee individuals in larger *V. uliginosum* populations (*F*_1,20_ = 19.43, *P*<0.001, Fig. 2a). The proportions of small and large bee species changed according to population size: large bumble bee species were relatively more abundant in large populations (F_1,19_ = 5.42, *P* = 0.03, Fig. 2b), whereas consequentially higher proportions of small bee species were observed in small plant populations (F_1,19_ = 5.64, *P* = 0.03).

Bumble bee species that were often absent or rare in small populations were particularly large ones such as *Bombus lapidarius* and *B. terrestris* OTU and the cuckoo species *B. campestris* and *B. vestalis* OTU (Table 2). However, concerning species richness of bumble bees, no relationship between the number of species and plant population size was found (*F*_ 1,20_ = 3.08, *P = *0.09). Also the diversity (Shannon H) of bumble bee assemblages did not depend on plant population size (*F*_ 1,20_ = 0.32, *P = *0.64).

Other fragmentation characteristics such as bog surface area (*F*_ 1,20_ = 0.92, *P = *0.35), population isolation (*F*_ 1,20_ = 0.66, *P = *0.43) and the proportion of surrounding forest (*F*_ 1,20_ = 1.87, *P = *0.19) had no significant influence on bee abundance or diversity.

Looking at the foraging behaviour of bumble bees, we noticed a change from pollen and nectar foraging in large plant populations to predominantly nectar foraging in small populations. On average, about 15% (range = 0 - 35%) of all bumble bees carried pollen loads. The percentage of individuals collecting pollen increased with plant population size (*F*_1,19_ = 8.82, *P* = 0.008, Fig. 2c). This result is most likely due to a change in foraging behaviour of large bumble bee species as hardly any individuals of the large bee species present in small plant populations were collecting pollen, i.e. the proportion of pollen foragers within the category of large species declined with plant population size (F_1,20_ = 5.66, *P* = 0.03). On the contrary, the proportion of small bumble bee species foraging for pollen remained high, irrespective of the plant population size (F_1,20_ = 0.01, *P* = 0.91, Fig. 2d).

### Visitor Fidelity

All but one of the pollen loads sampled contained *V. uliginosum* pollen, and pollen from this species was the dominant component (i.e. making up over 50% of the pollen grains in the load) in 28 of the 54 samples taken from *B. pratorum*. Other pollen species regularly visited for pollen were *Sorbus aucuparia* L. (dominant in 44% in all samples) and *Cytisus scoparius* L. (dominant in only four samples, all others <0.5%). Overall, 46% of the loads sampled were made up almost entirely (≥95%) of pollen grains belonging to one of these three plant species. Pollen from other plant species was rarely found (in 3 pollen loads with each <3.7%). Also virtually all (99%) pollen loads collected from other *Bombus* species contained only traces (<1%) of pollen from other plant species.

**Figure 3:**
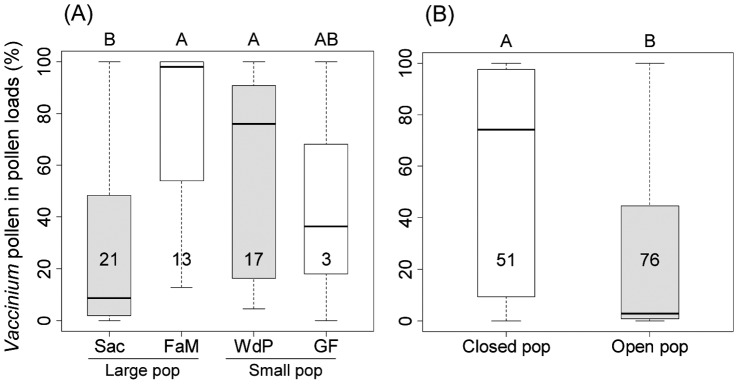
*Vaccinium* pollen (%) in corbicular pollen loads collected at different study sites. Data for *B. pratorum* (A) and all *Bombus* species (B) pooled. The sites are arranged from large populations (Sacrawé = Sac, Fange aux Mochettes = FaM) to small (Wé de Pourceaux = WdP, Grande Fange = GF); open, patchy populations are marked with grey boxes. Different letters indicate significant differences (*P*<0.05) according to Mann-Whitney-U tests (A) or GLMM (B). Figures in boxes stand for sample sizes. Standard box plots with lines = median, boxes = 25% and 75%, whiskers = minimum and maximum without outliers.

The proportion of *V. uliginosum* pollen in corbicular loads of *B. pratorum* varied significantly between different sites (Fig. 3a; χ^2^ = 13.22, d.f. = 3, *P* = 0.004). However, this variation seemed to be related to openness of the study site (proportion of surrounding forest) and/or shrub cover (Table 1), not to plant population size. When the pollen load samples from all bumble bee species were pooled, the average percentage of *Vaccinium* pollen per load did not differ significantly in large compared to small plant populations (F_1,118_ = 1.72, *P* = 0.192). However, at sites with 100% shrub cover and completely surrounded by spruce forest, i.e. Fange aux Mochettes and Wé des Pourceaux, bees showed a significantly higher fidelity to *V. uliginosum* than in sites that were more patchy, open and situated within a greater bog (Fig. 3b; F_1,118_ = 6.31, *P* = 0.013).

### Pollen Limitation of *Vaccinium uliginosum*

On average, 14.1 (±10.8) seeds per fruit were viable, which corresponds to a general seed set of 23.1% (±17.5%). Supplemental hand pollination did not result in higher seed set in any of the four investigated populations (“hand-pollinated” vs. “open-pollinated/same plant” and “open-pollinated/different plant”; Table 3). Only in the largest population (Sacrawé), was seed set significantly different between fruits resulting from the two types of natural pollination, i.e. those fruits growing on the same plants as the pollinated ones (“open-pollination/same plant”) had lower seed set than those growing on different plant individuals (“open-pollinated/different plant”; Table 3).

**Table 3:** Percentage (%) of viable seeds in fruits from different pollination treatments and study sites (mean [median]±SD).

Study site	Hand-pollinated	Open-pollinated/same plant	Open-pollinated/different plant	Test statistic (Kruskal-Wallis)
Wé des Pourceaux	28.3[23.3]±21.7	21.2[17.5]±15.3	18.8[18.5]±11.4	χ^2^ = 1.43, *P* = 0.489
Grande Fange	18.8[18.7]±10.8	13.8[11.1]±8.5	–	Z = 350, *P* = 0.061†
Fange aux Mochettes	25.4[19.8]±18.4	17.7[14.1]±13.1	20.7[15.7]±15.9	χ^2^ = 4.22, *P* = 0.121
Sacrawé	28.0[22.8]±21.1 ^AB^ †	22.8[16.7]±16.6 ^B^	34.2[29.2]±21.4 ^A^	χ^2^ = 7.41*, *P* = 0.025

† Mann-Whitney-*U.*

## Discussion

### Abundance and Diversity of Bumble Bees

Similar to other studies, we found a significant effect of plant population size on numbers of bumble bee individuals visiting flowers [44]–[46]. Thus, of all remaining fragments of *Vaccinium uliginosum* on the Plateau des Tailles, the smaller populations probably did not offer enough resources to support large numbers of bumble bees. This underpins the notion that bumble bees are especially prone to habitat fragmentation [47]. In spring, *V. uliginosum* is probably a crucial floral resource in the area, providing important nectar and pollen supplies. However, we did not find any impact of plant population fragmentation on either the species richness or diversity of bumble bees [21], [48]–[50], maybe because some of the small populations of *V. uliginosum* (e.g. Grande Fange, Chamfa, Table 1) were located in larger bogs providing other resources.

Our results showed that the abundances of large bumble bee species declined in small plant populations, and the proportion of small species increased. Large bee species were more sensitive to decreased floral resources, probably due to higher pollen requirements [12]. This is in contrast to the general perception that especially small-bodied bee species, associated with lower flight and dispersal abilities [16], are more susceptible to extirpation from small habitat fragments [19].

The consequences of habitat fragmentation on the landscape scale such as isolation neither affected the number of individuals nor species recorded in our study [49], [51]. Similar to Kreyer et al. [52], forest was no barrier to bumble bees, since we found no effect on bee abundance or diversity when sites were enclosed by forest. We conclude that population size (measured as plant surface area) is the most likely factor determining bumble bee occurrence. This is perhaps best illustrated by one large population that is most isolated from all others (Fange aux Mochettes, distance >4 km, 100% surrounded by forest, Table 1). In this population, both the abundance and number of bumble bee species were high for all observation dates (Table 2).

### Bumble Bee Foraging Behaviour

It has already been observed that the foraging behaviour of bees or other pollinators changes in small fragments [20], [21], [53]. However, although some studies concentrate explicitly on the diversity and abundance of pollen foraging bees [54], [55], we are not aware of other studies that simultaneously explore pollen and nectar foraging behaviour. Thus, a key result of our study is the change in bumble bee pollen collection behaviour: our analyses clearly show that in large *V. uliginosum* populations, significantly more bumble bee individuals were collecting pollen than in small populations. This tendency was induced by large sized species where individuals collected mainly nectar when plant population size decreased. On the other hand, individuals from small bee species continued to forage for pollen in small plant populations. Such a change in behaviour could compromise colony development and survival of larger species in small habitat patches.

*Vaccinium uliginosum* may be a good pollen resource for bumble bees. *Vaccinium* pollen has to be collected by buzzing the flower (sonication), which is thought to be a rapid and energetically efficient method to collect large amounts of pollen [56]. As for other sources of pollen which require buzz pollination (Ericaceae, Solanaceae, etc.), *Vaccinium* pollen is thought to be a high-energy source that is lipid- and protein-rich [56], [57]. Perhaps most importantly, most other flower visitors (e.g. *Apis* and syrphid flies) are not able collect *Vaccinium* pollen because they cannot sonicate the anthers. Interspecific competition for pollen should therefore be much lower than for nectar since no pollen is removed by these other visitors [12]. Nevertheless, due to the reduced number of flowers present in small populations, pollen supplies may be insufficient for larger species to continue collecting pollen. Bees also have to learn the skills how to handle such complex flowers and the respective time investment might only pay off at sites with abundant supplies [58]. According to Rasheed and Harder [59], bumble bees should forage on the most rewarding resource. In our study system, unprofitable pollen supplies in small populations could force bumble bees to switch pollen host or resource and collect nectar instead. Our observations show such an adapted behaviour and change to nectar collection for large bumble bee species.

### Bumble Bee Fidelity to *Vaccinium uliginosum*

Pollen load analyses indicated that *Bombus pratorum* was foraging exclusively on three plant species (*Vaccinium uliginosum*, *Sorbus aucuparia*, *Cytisus scoparius*). More than half of the pollen load samples were a mix of two species, hardly ever three. In contrast to nectar foraging, where bumble bees often switch between several plant species [60], it is known that bees are more specialised during pollen collection [61]. Especially for *B. pratorum*, a high degree of specialization during pollen collection is known [61], [62]. But also pollen loads from other bee species (*B. hortorum* s.l., *B. hypnorum, B. lapidarius, B. pascuorum*, *B. terrestris* s.l.) contained almost 100% pollen from the three mentioned plant species. This is perhaps more surprising since for these bumble bees, pollen from 13 (*B. pascuorum*) and nine (*B. terrestris*) different plant species have been reported in their pollen loads [62]. This suggests that the food web of bumble bees in the Upper Ardenne in spring is very limited. According to optimal foraging theory, individual foragers should enlarge their diet breadth when competition for one resource intensifies [63]. Our analyses of pollen loads did not confirm this hypothesis. Instead, fidelity to *V. uliginosum* as the main or only pollen source in corbicular loads seemed rather linked to patchiness and/or openness of the study site than to the size of the plant population. At sites that were totally covered with *Vaccinium* shrubs, and completely surrounded by spruce plantations, bumble bees foraged predominantly for *Vaccinium* (Fig. 3), even if attractive alternative species were flowering nearby (*S. aucuparia* and *C. scoparius* [64]). It is well known that pollinators forage with greater constancy when flower or plant densities are high [65], [66]. It could also be that in open sites, alternative plant resources might be more easily identified and reached by insects. For the investigated populations, openness coincided with shrub cover. Therefore, it was impossible to disentangle the influence of these two factors on bumble bee fidelity.

### Pollination Limitation of *Vaccinium uliginosum*

*Vaccinium uliginosum* is a self compatible species that also propagates via clonal growth [26], [67]. The flowers have characteristics that are linked to self pollination [68], but reduced fruit and seed set has been recorded in the absence of pollinators [69]. Our pollination experiments showed no sign for pollen limitation and there were no effects of plant population size on pollination success. The reproductive success of *V. uliginosum* therefore seems less sensitive to population fragmentation [17]. Nevertheless, outcrossing may be a rare event in small populations and genetic diversity might be lost through demographic, genetic and environmental stochasticity [70].

### Conclusion

Due to the limited number of plant populations we observed, we are careful not to make generalisations. However, the finding of changes in pollen collecting behaviour by bumble bees according to plant population size is novel and should receive further attention. Our observation that small bog fragments become unattractive for pollen collection by large bumble bee species may add to our understanding of why some bee species are more vulnerable to habitat fragmentation and what factors influence their coexistence [23]. We show that pollen load analyses offered relevant information about the composition of food webs. Such knowledge is fundamental in restored sites to evaluate conservation efforts [62]. Our results illustrate that *V. uliginosum* provides important resources for bumble bee species that are suspected to be in decline [47]. The plant species itself might not be at immediate risk of extinction. However, it is growing in an endangered habitat and populations should be subject to continued and intensified conservation practices in Europe. The connectivity of habitat fragments should be improved to offer a continuous web of resources for social bees.

## Supporting Information

Table S1 Identification of insect species visiting *Vaccinium uliginosum* flowers with total numbers of individuals observed from 2008–2010.
(DOC)
