# Supplementary material for: The Abundance and Pollen Foraging Behaviour of Bumble Bees in Relation to Population Size of Whortleberry (Vaccinium uliginosum)
Source: PLoS One. 2012 Nov 27;7(11):e50353. doi: 10.1371/journal.pone.0050353 (PMC3507688; doi:10.1371/journal.pone.0050353)
Supplement: Table S1 — Identification of insect species visiting Vaccinium uliginosum flowers with total numbers of individuals observed from 2008–2010. (DOC) [file pone.0050353.s001.doc]

**Table S1.** Identification of species visiting *Vaccinium uliginosum* with total numbers of individuals observed from 2008 - 2010.

| Order | Family | Genus | OTU | Total number of individuals | Determined species |
| --- | --- | --- | --- | --- | --- |
| Hymenoptera | Andrenidae | *Andrena* | Andrena | 84 | *A. cineraria* L.*, A. lapponica* Zetterstedt |
|  | Apidae | *Apis* | Apis | 54 | *A. mellifera* L. |
|  |  | *Bombus* | Campestris | 2 | *B. campestris* Panzer |
|  |  |  | Hortorum | 69 | *B. hortorum* L.*, B. jonellus* Kirby |
|  |  |  | Hypnorum | 58 | *B. hypnorum* L. |
|  |  |  | Lapidarius | 170 | *B. lapidarius* L. |
|  |  |  | Pascuorum | 242 | *B. pascuorum* Scopoli |
|  |  |  | Pratorum | 469 | *B. pratorum* |
|  |  |  | Terrestris | 361 | *B. terrestris, B. lucorum, B. cryptarum* Fabricius |
|  |  |  | Vestalis | 87 | *B. bohemicus* Seidl*, B. sylvestris* Lepeletier |
|  |  | *Nomada* | Nomada | 1 | *Nomada sp.* |
|  | Halicitidae |  | Halictus | 5 | *H. rubicundus* Christ*, Halictus sp.* |
|  |  |  | Lasioglossum | 8 | *Lasioglossum fratellum* Pérez |
|  | Vespidae |  | Vespa | 12 | *Vespa sp.* |
| Diptera | Syrphidae | *Eristalis* | Eristalis | 948 | *E. tenax* L.*, E. picea* Fallén |
|  |  | *Rhingia* | Rhingia | 199 | *R. campestris* Meigen |
|  |  | *Volucella* | Volucella | 82 | *Volucella sp.* |
|  |  | *Sericomyia* | Sericomyia | 10 | *Sericomyia lappona* L. |
|  |  | *Heliophilus* | Heliophilus | 10 | *Heliophilus pendulus* L. |
|  |  | *Chrysotoxum* | Chrysotoxum | 1 | *Chrysotoxum arcuatum* L. |
|  | Sarcophagidae | *Sarcophaga* | Sarcophaga | 3 | *Sarcophaga sp.* |
| Lepidoptera | Lycaenidae | *Callophrys* | Callophrys | 3 | *Callophrys rubi* L. |
|  |  | *Lycaena* | Lycaena | 1 | *Lycaena helle* Denis & Schiffermüller |
